# Reproductive biology and breeding system of *Saraca asoca* (Roxb.) De Wilde: a vulnerable medicinal plant

**DOI:** 10.1186/s40064-016-3709-9

**Published:** 2016-11-28

**Authors:** G. R. Smitha, V. Thondaiman

**Affiliations:** ICAR-Directorate of Medicinal and Aromatic Plants Research, Boriavi, Gujarat 387310 India

**Keywords:** *Saraca asoca*, IUCN, Conservation, Phenology, Reproductive biology, Pollination

## Abstract

Ashoka (*Saraca asoca*) is a perennial, evergreen tree valued for its ornamental flowers and medicinal values. This species is classified as ‘vulnerable’ under IUCN list due to its dwindling population because of destructive harvesting from natural habitats. Therefore, conservation and multiplication of this species is need of the hour to utilize its astonishing medicinal uses eternally. Conservation approaches of any plant species require in-depth study of its reproductive biology, which is lacking in this species. The present study is the first detailed report on reproductive biology of *S. asoca*. This tree bears fragrant flowers in paniculate corymbose inflorescence from December end to May, with peak flowering during February–March. The fruits attain its maturity during last week of May–July. Seeds were dispersed from the pod to the tree premises upon complete maturity. The time of anthesis in this species is noticed in the early morning from 3.00 to 5.30 am, which coincided with anther dehiscence, stigma receptivity and insect activity. The length of the stamen and pistil points towards the pollination compatibility in both male and female parts. Pollen viability was maximum within 2 h of anthesis, which decreased thereafter and no pollens were viable after 6 h. The stigma was receptive at the time of anthesis and continued for 24 h. The tree produces bright colour attractive flowers, which changed from yellow/light orange to scarlet/red from the inception of buds to wilting. The bright color of the flowers attracted floral visitors/pollinators thereby facilitated the pollination in this species. The observations of the floral biology and breeding system indicated the cross pollination behaviour, which limited the production of selfed seeds and would help to maintain the sustainable levels of heterozygosity among the various populations. Considerable amount of seeds produced in this species indicated that the species is capable of sustaining its progenies in the natural populations. Polyembryony to an extent of 5% was also recorded in this species.

## Background

Ashoka (*Saraca asoca* (Roxb.), De. Wild) belonging to family Fabaceae is one of the most legendary and sacred tree of India. It is commonly called as *Ashoka*, *Sita ashoka*, *Karkeli* (Sanskrit), *Sita ashok*, *Ashoka* (Hindi, Bengali, Gujarati, Marathi), *Asogam* (Tamil), *Oshok* (Bengali), *Ashokmu*, *Vanjulamu* (Telugu), *Asokam* (Malayalam), *Alshth*, *Achenge*, *Kenkalimara*, *Ashokadamara* (Kannada). The word *Ashoka* literally means “without sorrow”, a reference to this bark’s reputation for keeping a woman healthy and youthful (Nayak et al. 2009; Pradhan et al. 2009). It is distributed in Central and East Himalaya, East Bengal, Burma, Western Peninsula, Ceylon and Malaya. In India, it is found in the foothills of central and eastern Himalayas, northern plains as well as in west coast of the subcontinent (Preeti et al. 2012). It is also cultivated in tropical gardens as an ornamental tree for its evergreen beautiful foliage and attractive flowers. Apart from this, the tree also has many health benefits and has long been used in traditional Indian medicine as a key ingredient in various therapies and cures. This tree is closely associated with Indian cultural traditions. Bark, flower, leaves, roots and seeds of ashoka are used as medicine. The bark contains tannin, catechol, flavanoides, sterol, glucosides, alkaloids, other organic calcium compounds (Kirtikar and Basu 1981), which contributes to its medicinal qualities as ‘female tonic’. Bark is extremely useful in gynecological problems, especially in the treatment of menstrual disorders associated with excessive bleeding, congestion, pain, dysmenorrhea, abdominal pain, and uterine spasms. It is also used for leucorrhea and has an astringent but stimulative effect on endometrium and ovarian tissues (Nadkarni 1976; Nudrat and Usha 2005). The bark is used to cure dyspepsia, dysentery, piles, sores and irregular menstruation, whereas the dried flowers are used for treatment of syphilis, hemorrhagic, diabetes and dysentery. It also helps to get rid of the toxins from the body and is effective in purifying the blood naturally and in preventing skin allergies. Seeds are used to treat bone fracture and vesicle calculi. The plant is used in the treatment of dyspepsia, indigestion, blood disorders, tumours etc. (Kirtikar and Basu 1981). There are various indigenous preparations available with Ashoka as a major constituent, of which the important ones include *Ashokarishta*, *Ashokaghrita*, *Ashoka kwath* (Ghose 1984; Pradhan et al. 2009).

The indiscriminate use and destructive harvesting process of ashoka bark has led to acute scarcity of the genuine raw drug and this in turn, has led to cost escalation and widespread adulteration or substitution (Beena and Radhakrishnan 2010; Pradhan et al. 2009). This has resulted in dwindled population in wild and the species has been included in the ‘vulnerable’ category of the ICUN list (IUCN 2011). The unscientific management practices, ever increasing demand for its bark and phytochemicals, poor seed viability (Pushpagandhan et al. 2004) and over-exploitation of the plant parts like bark, flowers, seeds etc. has contributed greatly for the declining population of the species. Understanding the reproductive biology of this tree would help in developing effective strategies for its sustainable utilization and establishing suitable plus tree breeding programmes for improvement of the existing population. The studies can also help in developing certain protocols to combat the problems that impede species regeneration (Moza and Bhatnagar 2007).

In recent past, a number of scientific studies have been conducted to promote cultivation of medicinal plants to reduce the dependence on wild stock. A few studies have also been conducted in *S. asoca*. A study on karyomorphological analysis revealed the presence of 34 chromosomes in the somatic cells of *S. asoca* (Deepa et al. 2013), in contrast to the earlier report of 2n = 24 (Singh 2002; Singhal et al. 1990). Flowering behaviour of mature ashoka trees was studied at Trissur (DMAPR 2008, 2009). Smitha (2013) standardized different methods of vegetative propagation of Ashoka through air layering and grafting for multiplication of superior quality, true-to-type trees. Rama Subbu et al. (2008) standardized in vitro clonal propagation of *S. asoca* using shoot tip, nodal and internodal explants, wherein the frequency of shoot organogenesis was highest (82%) in 0.5 mg/l benzylaminopurine (BAP) and microshoots rooted well on Murashige and Skoog (MS) medium supplemented with 4.0 mg/l of IBA. High frequency induction of friable callus using sections of hypocotyls, epicotyls and young leaves on full and half strength MS medium supplemented with BAP and NAA has been reported (Waman et al. 2010). A protocol for callus development has also been standardized at DMAPR, Anand, wherein the frequency of callus induction was higher in young leaves and nodal explants as compared to the immature inflorescence (DMAPR 2014). Recently, ‘Aswani-1’ the first variety of ashoka, has been released from All India Coordinated research Project on Medicinal and Aromatic Plants and betelvine (AICRP on MAP & B), Vellanikkara, Trissur through single plant selection. This variety is reported to possess high bark yield (2.8 kg/plant dry bark), thick bark (7 mm) with 3.3% tannin content. There are few reports available for polyembryony in ashoka (Waman and Bohra 2013; Wanage et al. 2010).

 Some researchers have paid attention to the reproductive biology of medicinal plants. Systematic studies on reproductive biology have been carried out in economic medicinal plants such as *Aconitum balfourii* (Nautiyal et al. 2009), *Aloe vera* (Rathod et al. 2014), *Chlorophytum borivilianum* (Geetha and Maiti 2001), *Commiphora wightii* (Arn.), Bhandari (Kawane et al. 2015), *Inula racemosa* (Wani et al. 2006), *Psychotria ipecacuanha* (Souza et al. 2008), *Merremia macrocalyx* (Raimundez-Urrutia et al. 2008), *Plumbago zeylanica* L. (Abera et al. 2008), *Stachytarpheta maximiliani* (Barbola et al. 2006) etc. However, no systematic report on reproductive biology of ashoka is available so far. Keeping the above facts in view, a detailed study of the reproductive biology of ashoka growing at various places of Anand, Gujarat has been undertaken for three years period. The present study will unravel the critical events, which in turn will help in planning strategies for effective and efficient conservation, and management. This would also pave the way to a sustained generation of raw material for commercial usage in pharmaceutical industries in future, and regeneration for habitat enrichment.

## Methods

*Saraca asoca* is a medium sized, evergreen tree grows to a height of 7–10 m, which occurs up to an altitude of 750 m MSL. Leaves are 15–20 cm long, rachis glabrous, corky at the base; petioles very short; stipules intrapetiolar, completely united, 4–6 pairs of leaflets which are oblong- lanceolate, obtuse or acute, quite glabrous, base rounded or cuneate. The bark is rough and uneven, dark brown or grey or almost black with warty surface. Bark channeled, smooth with circular lenticels and traversely ridged, sometimes cracked (Ali 2008; Rastogi 2003).

The botanical classification of this species is as follows (Pradhan et al. 2009);

- Kingdom: Plantae
- Divison: Magnoliophyta
- Class: Magnoliopsida
- Order: Fabales
- Family: Fabaceae (earlier: Caesalpinaceae)
- Genus: *Saraca*
- Species: *asoca*

### **Study site**

The studies were conducted for three consecutive years (2012–2013, 2013–2014 and 2014–2015) at research farm of Indian Council of Agricultural Research-Directorate of Medicinal and Aromatic Plants Research (ICAR-DMAPR), Boriavi, Gujarat, India located at 22.5°N latitude and 73.0°E longitudes. Ten trees each were marked at three locations within the ICAR-DMAPR premises viz., Boriavi farm, Lambhvel farm and DMAPR residential complex. The trees undertaken for this study were eight to nine years old.

### **Phenology**

Phenological events such as bud break, flowering period, peak flowering period, time taken for flowering, fruit set and maturity were recorded from ten labeled trees each at three locations. To obtain the above flowering traits, observations were made every day in the morning throughout the flowering season, while for fruit traits, observations were made once in a week (Tandon et al. 2003).

### **Quantitative and qualitative traits of flower**

The average numbers of inflorescences per tree were recorded in all the three locations (n = 10 trees) and average flowers borne on an inflorescence were recorded from a set of randomly tagged inflorescences (n = 10 per tree). Number of bisexual flowers per inflorescence, percentage of bisexual flowers, fruit set and number of fruits per inflorescence were recorded at appropriate stages of growth and development. Flower color (scored flower color with the standard color chart (SCC), the Royal Horticultural Society, London, England) and stamen length, stigma length and corolla tube length were studied in ten trees (n = hundred flowers per tree) following methodology of Kaufman et al. (1989), Lawrence (1951) and Nath (1996). Randomly selected 20 flowers from each study site were selected to carry out the flower study in terms of both qualitative and quantitative traits.

### **Breeding system**

To investigate the nature of breeding system operative in this species the following aspects were studied:

### **Anthesis and anther dehiscence**

Mature flower buds in twenty inflorescences were tagged and the time of anthesis was observed over a period of four days at different intervals (3 am, 8 am, 3 pm and 8 pm). The same flowers were used for recording the time of anther dehiscence (Nautiyal et al. 2009).

### **Pollen: ovule ratio**

For pollen count, the anthers were collected from mature flower buds from each tree prior to anther dehiscence and were crushed to release the total pollens in 0.5 ml of distilled water along with a drop of Tween-20. The solution was homogenized and pollen count was done by using Haemocytometer (Waller et al.^1998^). Average number of ovules per pistil was counted using dissection microscope.

Pollen–ovule ratio was calculated following Cruden’s (1977) method as follows:

$${\text{P}}/{\text{O}} = \frac{{{\text{Pollen}}\;{\text{count}}\;{\text{per}}\;{\text{anther}} \times {\text{No}}.\;{\text{of}}\;{\text{anthers}}\;{\text{per}}\;{\text{flower}}}}{{{\text{Number}}\;{\text{of}}\;{\text{ovules}}\;{\text{per}}\;{\text{flower}}}}$$

### **Pollen viability and pollen germination**

Pollen grains freshly collected from 20 flowers were taken and viability test was carried out using Fluorochrome Reaction (FCR) test as per the method proposed by Heslop-Harrison and Heslop-Harrison (1970). Observations were taken under fluorescence microscope (Olympus BX 50) under UV excitation. Pollen grains showing fluorescing green were considered as viable and data were presented in percentage. To study the pollen germination, the pollens were collected at the time of anthesis (3 am) from marked trees and were immediately put in sucrose solution and Brew Baker’s solution of different concentrations (0.1, 1, 2, 3, 4, 5, 10, 15, 20 and 25%). The culture were kept in incubator and observed for pollen germination at constant intervals (2, 4, 6 and 8 h after anthesis). The pollen germination was observed using a microscope (Brewbaker and Kwack 1963).

### **Stigma receptivity**

Receptivity of stigma was analyzed with H_2_O_2_ method (Dafni 1992), wherein bubbling in presence of hydrogen peroxide is considered as a positive result (Osborn et al. 1988).

### **Breeding behavior**

As described below, different pollination mechanisms were studied during this investigation. The pollination studies were conducted in randomized block design consisting of five treatments with three replications. The data pertaining to the fruit set percentage in different breeding methods was tabulated and statistically analyzed using statistical software SPSS. The pollination studies were examined across the five treatments using analysis of variance (ANOVA) and comparison between the treatment means was done at 5% probability level. The standard error of mean was calculated for each treatment using three replications and presented as ±*SE.*

(i) *Natural pollination* unopened buds on a plant were selected, tagged and left as such for natural pollination; (ii) *Selfing (autogamy)* unopened individual flowers in an inflorescence were selected, tagged and covered with butter paper to prevent out crossing. The bags of the selfed flowers were removed only after fruit development stage; (iii) *Geitonogamy* entire inflorescence was bagged before flower opening; (iv) *Open pollination* the unopened buds were carefully opened by removing petals and exposing the anthers. These anthers were carefully removed with the help of fine forceps without injuring any part of the flowers. These emasculated buds were then tagged and left as such. All the surrounding buds/flowers were removed to minimize the chances of geitonogamy. (v) *Cross pollination* The mature buds about to open next day were emasculated and bagged. The bags were opened on the day of anthesis and the flowers manually pollinated by gently brushing the receptive stigma with freshly dehisced anthers after which the flowers were re-bagged. During this, pollen grains were used from different trees (Tandon et al. 2003). Fruit set percentage was calculated by counting the number of fruits produced in the treatment (Shah et al. 2011).

## Results

### Botanical description

#### Phenological studies

**Fig. 1:**
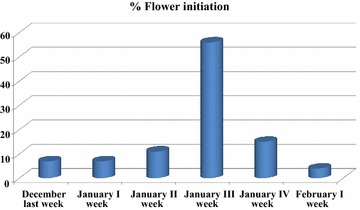
Ashoka flowering in different months under Anand locality

The observations on season of flowering in all the three years data showed that the inception of flowering primordial in the leaf axils of ashoka occurred during early December. The flower buds were yellowish green and commenced opening from December end and continued until May (Fig. 1), with peak flowering between February and March. The trees remained in bloom for 2–3 months. Fruiting commenced from March end to July with peak during May–June. The fruits attained maturity from last week of May to July. Seeds dispersed from the pod on the ground upon complete maturity.

#### Floral biology

**Fig. 2:**
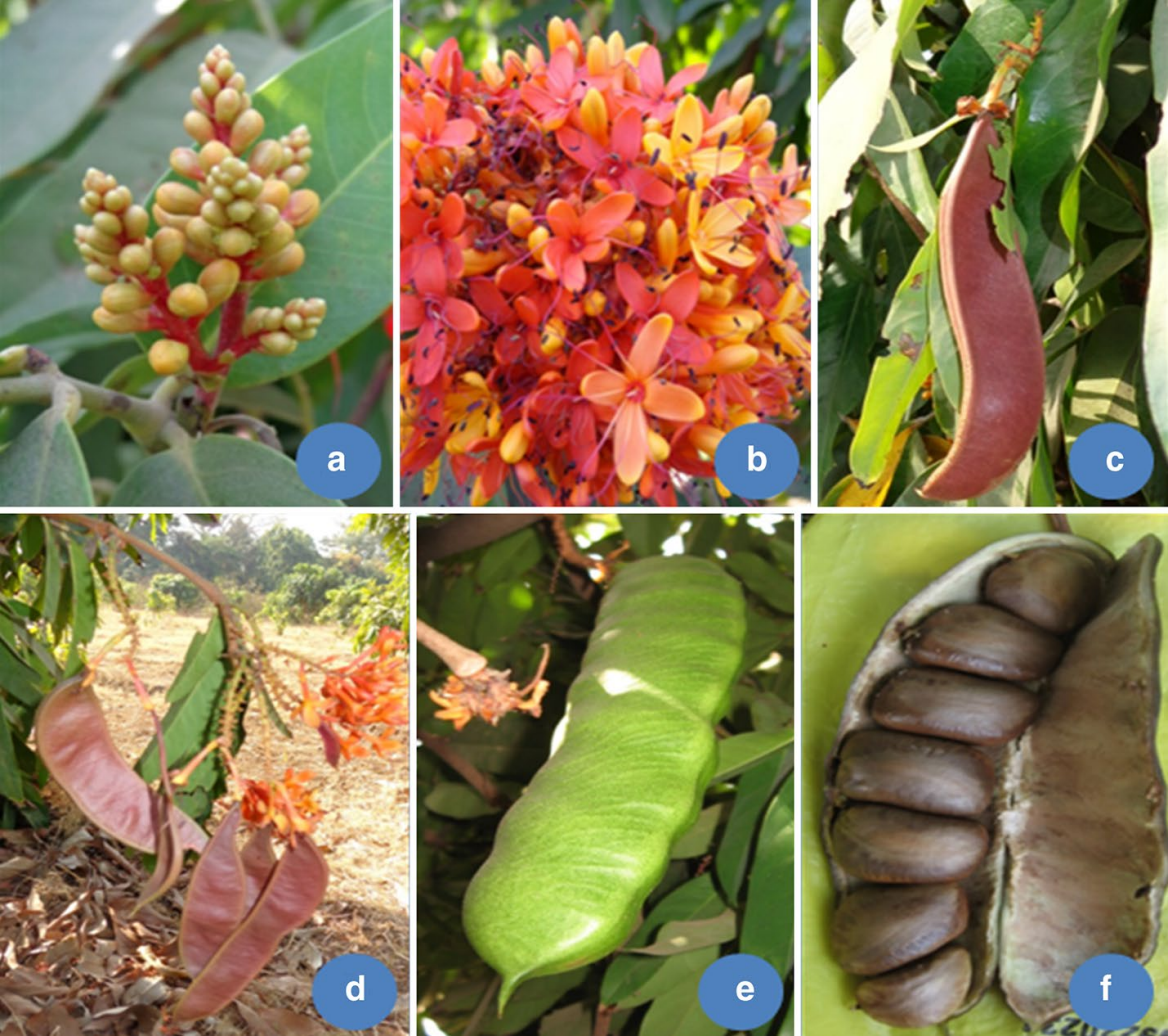
Stages of flowering and fruit development in *S. asoca*. **a** Flower bud, **b** inflorescence in full bloom, **c** pod setting, **d** pod development, **e** pod maturation, **f** seed dehiscence

**Fig. 3:**
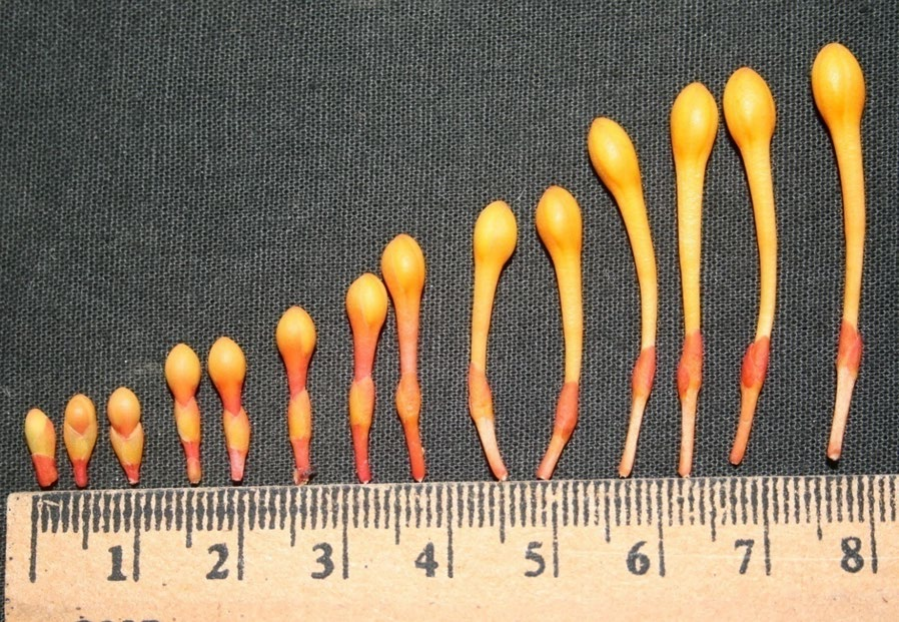
Sequence of flower bud development in *S. asoca*

**Fig. 4:**
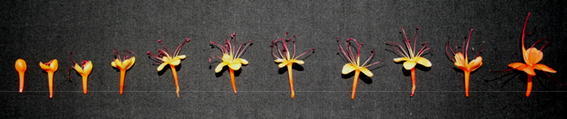
Sequence of flower opening/anthesis in *S. asoca*

Ashoka tree bears fragrant flowers in heavy, lush bunches. Petals were partial gamopetalous, in the form of corolla tube, separate on upper end, four in number. The flowers arranged closely in a cluster on every branch and twig, each cluster consisting of many small, long-tubed flowers opening out into four oval lobes. In an inflorescence, flowers open in acropetal succession and undergo anthesis in the same order. Both hermaphrodite and staminate flowers were present in same inflorescence (Fig. 2). In general, there were seven stamens, with some exceptions in which eighth were rudimentary. Rudimentary androecium were also present in few flowers that were normal than the size of normal androecium. Similarly, in some flowers, rudimentary ovary was also noticed. The length of the stamen ranged from 1.80 to 3.20 cm with the mean value of 2.51 ± 0.26 cm, pistil length ranged from 1.5 to 3.5 cm with mean value of 2.88 ± 0.32 and corolla tube length ranged from 1.10 to 3.00 cm with the mean value of 2.08 ± 0.29 cm. The flowers were yellow/light orange when young and became orange/scarlet, then crimson with age, before turning red at wilting (Figs. 3, 4). The flower colour at different stage of anthesis was compared with the Colour chart of Royal Horticultural Society (RHS)—Kew, London and presented in Table 1.

**Table 1:** Colour variation in flowers of Ashoka during different developmental stages

Flower stage	Group	No.
Bud	Yellow orange	23B
0 DAA	Orange	24A
1 DAA	Orange	25A
2 DAA	Orange red	30B
3 DAA	Orange red	N34B

*DAA* days after anthesis

On an average, a tree produced 43.40 ± 5.42 inflorescences, with an average of 43.56 ± 14.62 flowers/inflorescence and 1890.30 ± 150.92 flowers/tree. Hermaphrodite, staminate and pistillate flowers were observed either in the same inflorescence or in different inflorescences of the same tree. Among the total flowers, the number of hermaphrodite flowers per tree was counted as 564.90 ± 53.95, which was 29.97% of the total flowers per tree. The mean number of fruits per tree was recorded as 38.80 ± 9.77, which amounted to 2.05% fruit set of total flower production (Table 2).

**Table 2:** Quantitative flower characters of *S. asoca* (pooled data of three years)

S. no.	Characters	Observations
1.	Total number of inflorescences per tree	43.40 ± 5.42
2.	Number of flowers per inflorescence	43.56 ± 14.61
3.	Total number of flowers per tree	1890.30 ± 150.92
4.	Number of hermaphrodite flowers per tree	564.90 ± 53.95
5.	Percentage of hermaphrodite flowers	29.97 ± 2.86
6.	Stamen length	2.51 ± 0.26
7.	Pistil length	2.88 ± 0.32
8.	Corolla tube length	2.08 ± 0.29
9.	Number of fruits per tree	38.80 ± 9.77
10.	Percentage of fruit-set	2.05 ± 0.47

The values in the table are the mean for different variables ±*SEM*

#### Time of anthesis and anther dehiscence

It was observed that the flower started opening at 3 am and completed at 5.30 am (Fig. 4). Maximum flowers per inflorescence were opened during 3.00–5.30 am, which accounted to 98.90% of the total. While, very few flowers opened after this i.e. till 8 am. No flower was found opened during rest of the day. Anther dehisced at the time of anthesis.

#### Pollen biology

**Fig. 5:**
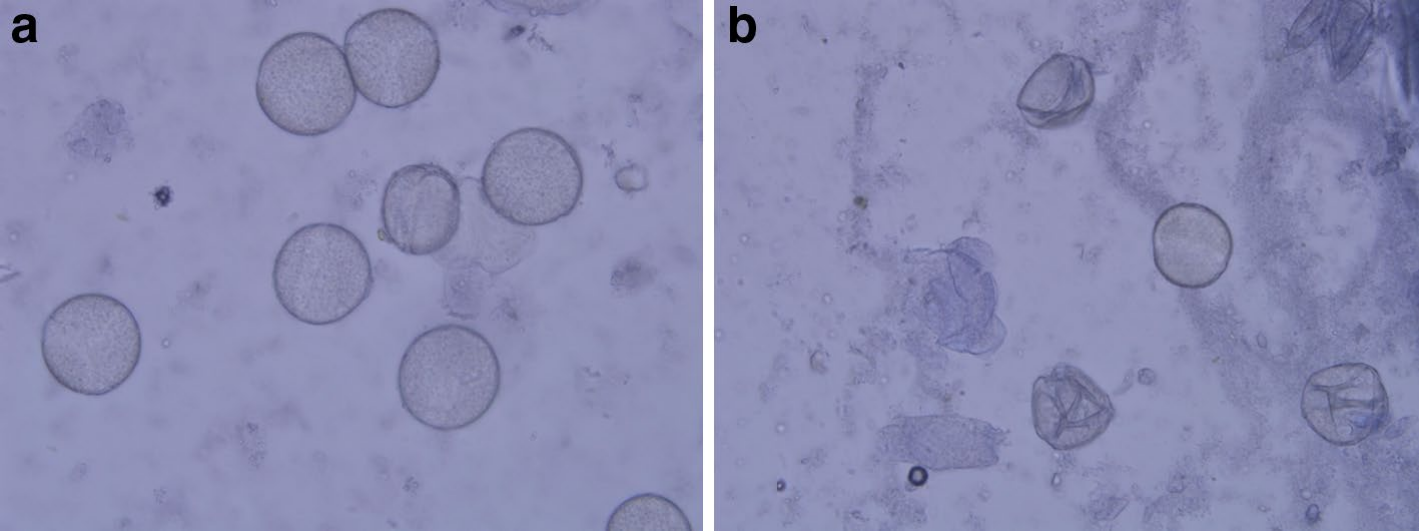
Pollen viability in *S. asoca*. **a** Viable pollens, **b** non viable pollens

**Fig. 6:**
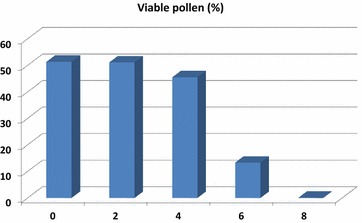
Pollen viability of *S. asoca* flowers at different hours after anthesis

 The pollen grains were oval to spherical in shape. Ashoka trees produced large number of pollens per flower. The number of pollens per flower was 14,399.00 ± 1710.84. Pollen viability test was carried out at different timings from anthesis. At the time of anthesis, 51.34% pollens were found viable and thereafter pollen viability was tested with 2 h difference. It remained almost the same (51.12%) after 2 h after anthesis (Figs. 5, 6). The pollen viability reduced to 45.48 and 13.38% after 4 and 6 h of anthesis, respectively. No viable pollen was recorded 8 h after anthesis. Hence, pollens collected immediately within 2 h of anthesis were used for pollination studies. No germination of pollens was observed in any of the above concentrations. The number of ovule produced by the flower ranged from 4 to 12 with the mean value of 7.96 ± 1.93. The pollen: ovule ratio worked out was 1808.92:1.00.

#### Stigma receptivity

**Fig. 7:**
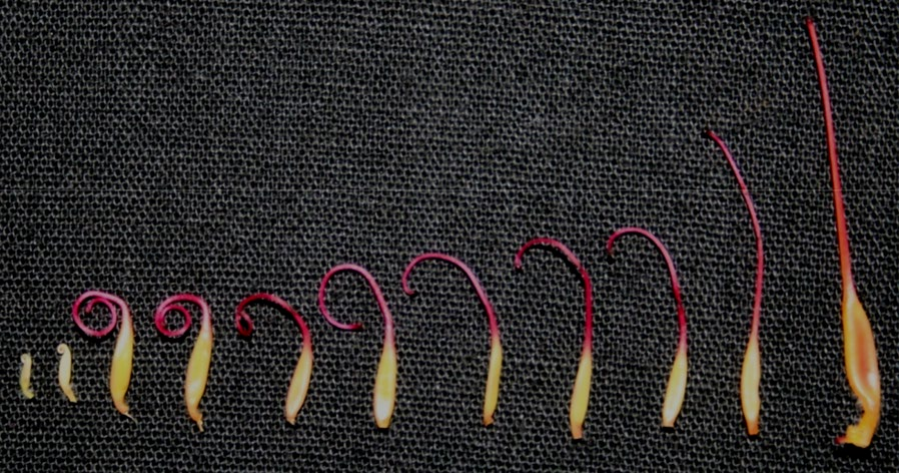
Sequence of stigma development in *S. asoca* flowers from bud to anthesis

**Fig. 8:**
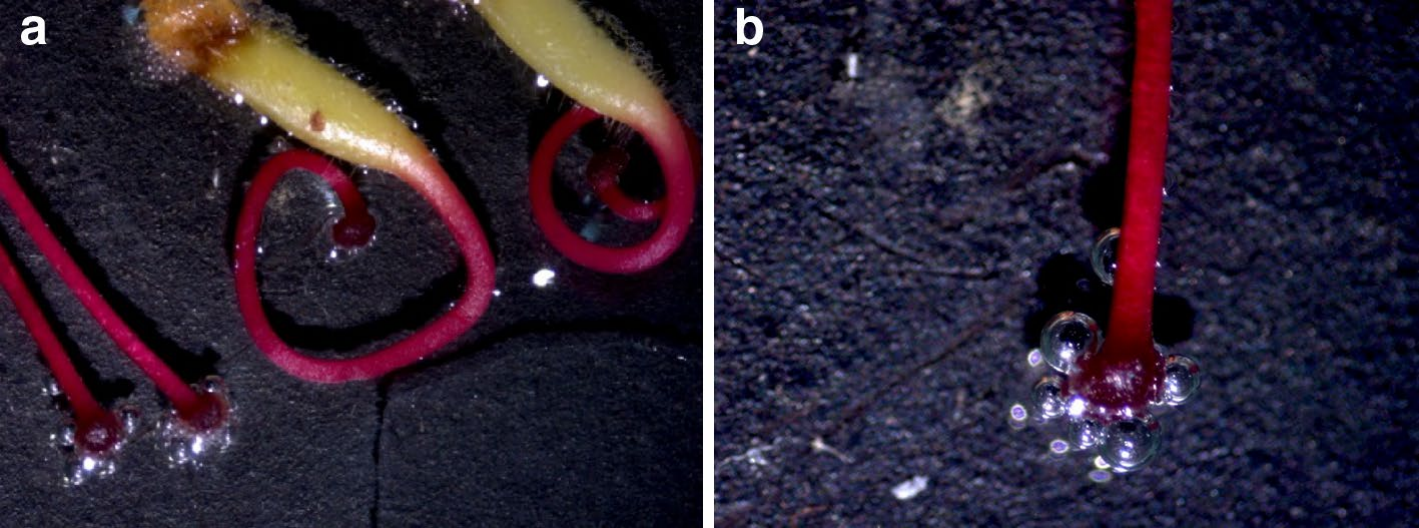
Stigma showing receptivity through bubbling in H2O2 test. **a** Four different stages of stigma development, first stage is not showing any bubbling, while rest three showing bubbling. **c** Close up view of third stage stigma showing bubbling to H2O2 test

To study the receptivity of stigma, the style was divided into 11 stages based on their maturity and developmental stage in the flowers (Fig. 7). The stigmas of initial stage i.e. fully curled with one or two loops did not showed any receptivity. Style just started unfurling during the initiation of the anthesis i.e. stage 4, and thereafter style showed bubbling, when treated with H_2_O_2_, confirming stigma receptivity at these stages (Fig. 8). The stigma was receptive at the time of anthesis up to 24 h after anthesis. This was also confirmed by the emasculation and hand pollination experiments at different intervals.

#### Pollination studies

In order to understand the breeding system operative in these species, different pollination systems like autogamy, geitonogamy, xenogamy, open pollination, cross pollination were carried out (Table 3). The data were subjected to analysis of variance (ANOVA) and inferences were drawn. The statistical test showed significant size difference (p = 0.05) among different pollination methods. Significantly highest fruit set was recorded in cross pollination (2.43%), compared to autogamy. However, there was no statistically significant differences were observed between natural pollination (2.05), open pollination (2.28) and cross pollination (2.43) and they all are at par with each other, but, they recorded statistically highest fruit set compared to geitonogamy (0.46). Interestingly, there was no fruit set observed in individual flowers bagged before anthesis to self-pollinate (autogamy); whereas the individual inflorescences bagged for controlled geitonogamy recorded 0.46% fruit set. The species showed maximum fruit set in allogamy and open pollination compared to autogamy and geitonogamy (Table 3).

**Table 3:** ANOVA of fruit-set percentage in different pollination methods (pooled data of three years in ten trees in three locations) of *S. asoca*

Types of pollination	No. of flowers studied	Fruit set %
Natural pollination	2320	2.05^a^
Open pollination	286	2.28^a^
Self pollination (autogamy)	227	0.00
Geitonogamy	7286	0.46^b^
Cross pollination	318	2.43^a^
F value		7.97*
SE (m)		0.44
Standard deviation (SD)		1.24
CD (p = 0.05)		1.32
CV (%)		16.11

*SE* (*m*) standard error of mean

* Significant at P ≤ 0.05; the values are the mean for different variables ±*SE*

Same letter in the superscript (^a^) does not differ statistically

#### Floral visitors

**Fig. 9:**
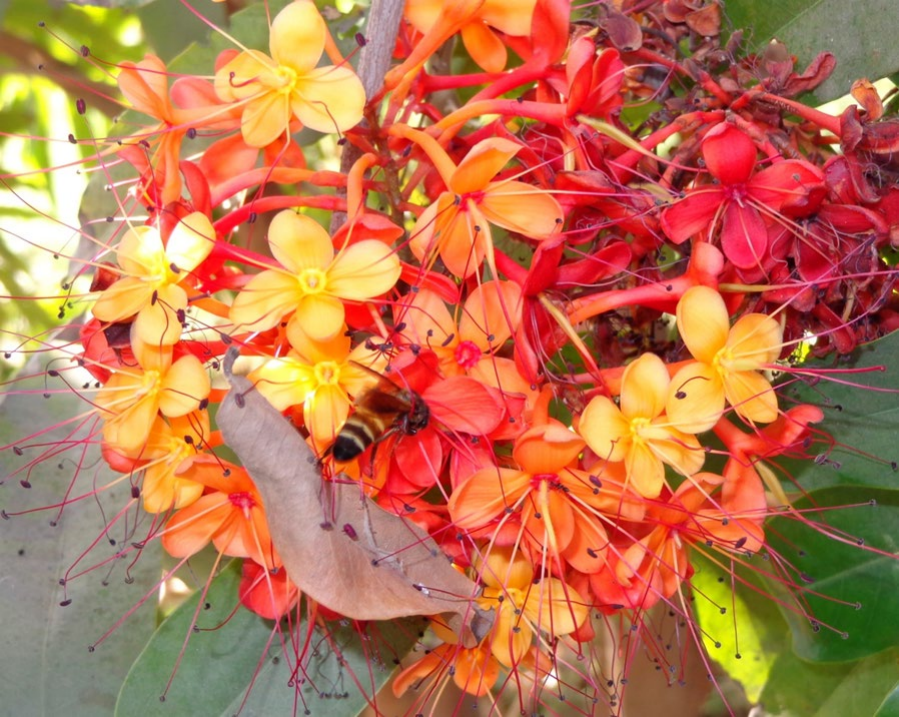
Giant honey bee visiting ashoka flowers

Freshly opened flowers were bright orange yellow in colour and attracted large number of insects; most of them from bee family besides black ant (*Oecophylla smaragdina*), syrphid fly (*Ischiodon* sp.), Arctidae moth, white ants and butterflies (Sulphides). Maximum visits occurred between 9 am to 12 pm, which coincided with pollen and stigma receptivity. Nectar produced in minute quantities constituted primary floral reward for the visitors, which was approximately 2.33 µl per flower. Giant Asian honeybee (*Apis dorsata*) was the most common visitors among the bee family and other small bees also visited the flowers regularly (Fig. 9). It visited many flowers in different inflorescence at a time.

#### Fruit setting and seed development

It took around two months from flowering to complete seed maturity. Different stages of pod development have been presented in Fig. 2. The seeds are bold, ovoid to ellipsoid in shape, flat, shiny, hairless and covered with a brown thin seed coat with two to eight seeds per pod (Fig. 2). On an average, 1 kg accommodated 160 seeds. Test weight of hundred seeds ranged from 633.00 to 969.00 g. Seed size was highly variable, which ranged from 2.06 to 11.56 g. Average length and breadth of the seeds varied from 2.80 to 5.80 cm and 1.60 to 4.10 cm, respectively.

#### Occurrence of polyembryony

Ashoka seeds were observed with poly-embryonic nature having 2–4 seedlings/seed. To record the percentage of polyembryony, the seeds were collected from 37 trees of Anupam mission and DMAPR campus (Boriavi and Lambhvel farms) and kept for germination. Among 454 seeds germinated, 23 were polyembryonic, which accounted to 5.07%. Further, it was also observed that the polyembryony phenomenon was tree-specific.

## Discussion

Considering the ever increasing demand for phytochemicals present in the plant parts of *S. asoca* and its dwindling population in the wild, development of suitable conservation strategies is the only alternative to utilize its astonishing medicinal properties. Any conservation approach requires an in-depth study of the phenology and reproductive biology (Moza and Bhatnagar 2007). The phenological studies in general and flowering in particular are useful in planning the conservation strategies as well as formulating measures for cultivating such species on large scale (Bernardello et al. 2001). *S. asoca* has spread in S.E. Asia, but in our study, we have recorded the flowering season and duration of this important species in three nearby localities of Anand, Gujarat condition mainly to initiate breeding work in this crop. Apart from genotype/ecotype, phenology of tree species depends upon different environmental factors like temperature, precipitation, elevation, soil water availability, day length etc. Hence, this work can be further studied in wider environmental conditions to study the variation in phenology and reproductive biology of this species.

Generally, the Ashoka tree starts flowering from three to four years after planting and the substantial number of flowers and fruit setting was noticed only after 6–7 years. Our study revealed that under Anand locality, flowering was noticed only from December to May months with the peak flowering during February to March; similar is the case in Trissur locality of Kerala State. However, under Trissur condition, trees produced flowers at least in some of the branches throughout the year, except during September and October months. Maximum matured pods were recorded during April followed by March which requires two months from flowering to complete seed maturation. Number of seeds per pod was also higher during February to April (DMAPR 2008, 2009).

The tree produced fragrant, bright coloured attractive flowers, which changed its colour from yellow/light orange to scarlet/red from the inception of buds to wilting. The bright color of the flowers attracted floral visitors/pollinators, which facilitated the pollination in this species. Similar finding was reported by Tandon et al. (2003) in *Butea monosperma.* Anthesis and anther dehiscence is most important event in the process of flower development. The time of anthesis in this species noticed in the early morning from 3.00 am to 5.30 am, which coincided with anther dehiscence and stigma receptivity. The length of the stamen and pistil points towards the pollination compatibility in both male and female parts. The number of pollens per flower, pollen: ovule ratio and pollen viability revealed the sufficiency of the pollen traits for its fertilization. In the pollen germination study tried with several concentrations of sucrose and Brew Becker’s medium, no pollen germination was observed in any of the years studied. It may be due to the refined morphological and functional characteristic of the stigma (Aronne et al. 2012). The anatomical feature of the sexual organs viz. pollen and stigma could further be studied thoroughly in this species to understand the pollen germination process (Arceo-Gomez et al. 2011).

Pollination behavior is required to be studied for conservation and crop improvement programme, as genetic variations are present. Pollination success in plants is determined by the timing of flowering, anther dehiscence and stigma receptivity (Renata et al. 2006). The results of this study on floral biology and breeding system indicate the reproductive potential of the species for cross pollination, which would limit the production of selfed seeds and as such is likely to maintain the sustainable levels of heterozygosity among the various populations. The results obtained from pollination studies revealed predominant self-incompatibility in this species as there was no fruit set in self-pollinated flowers (Borges et al. 2009). Comparison of all the pollination methods studied revealed the prevalence of cross pollination in this species. The fruit set observations in natural condition was recorded as 2.05%. Among the different pollination methods tried out more percentage of fruit set was found in manual cross pollination (2.46%), which was comparable with open pollination (2.28). The geitonogamous pollination method acquired only 0.46% of fruit set. This bespeaks the high degree of cross pollination in this species, while comparing to self pollination (autogamy and geitonogamy). Similar kind of geitonogamy and xenogamy has been observed in other species of Fabaceae, *Pterocarpus santalinus* Lf. (Rao and Raju 2002).

The presence of nectar constitutes the primary floral reward for the visitors and bright color of the flower attracts large number of visitors. Study on floral visitors concluded that honey bees are the major floral visitor facilitating pollen transfer (Tandon et al. 2003). Seeds of Ashoka were observed to be poly-embryonic to an extent of 5.07%, which is tree specific. Wanage et al. (2010) also reported polyembryony in *S. asoca* to an extent of 5.13%, of which 2.56% of plant showed triplet seedlings and remaining had four and five seedlings from single seed, which contribute to about 1.28%, each. Such observations on occurrence of polyembryony at seedling stage have already been reported in several tropical tree species such as *Dalbergia sissoo* (Kumar et al. 1977), *Bombax ceiba* (Venkatesh and Emmanuel 1978), *Putranjiva roxburghii* (Thapliyal 2004), *Nothapodytes nimmoniana* (Hombe Gowda et al. 2004), *Mangifera indica* (Kannur et al. 2005), *Garcinia indica* (Gunaga and Vasudeva 2008a), *Mammea suriga* (Gunaga and Vasudeva 2008b), *Humboldtia vahliana* and *Syzygium mundagam* (Jose et al. 2009). The genetic potential of such abnormal seedlings, if desirable can be used for future breeding programmes (Wanage et al. 2010). Since, epicotyls and hypocotyls were found to be useful in production of friable callus in ashoka (Waman et al. 2010), the polyembryonic seedlings could help in rapid multiplication of true-to-type plants through in vitro means (Waman and Bohra 2013).

## Conclusion

Reproductive biology is an important interdisciplinary area of plant sciences, which is essential to understand the evolution and survival of the species, to develop effective conservation strategies for exploitation of the economic potential of the medicinal plant species. In view of the above, reproductive biology and pollination behavior (flowering phenology, pollination types, role of pollinators) of *S. asoca* was studied under ex situ conditions. Our studies revealed that this species is an out-breeder. Fair number of seeds are produced by this species indicates that the species is capable of sustaining its progenies in the natural populations. However, the reason for the species to reach ‘vulnerable’ stage may be due to unscientific harvesting of its plant parts and poor seed viability and recalcitrant nature of the seeds (Smitha and Das 2016). The phenological and flowering studies could be helpful in setting up the conservation strategies as well as formulating measures for crop improvement and sustainable cultivation of this vulnerable species.
